# Correction: Goodbye to the bioassay

**DOI:** 10.1039/c8tx90016g

**Published:** 2018-07-31

**Authors:** Jay I. Goodman

**Affiliations:** a Department of Pharmacology and Toxicology , Michigan State University , East Lansing , MI 48824 , USA . Email: goodman3@msu.edu ; Tel: +1-517-353-9346

## Abstract

Correction for ‘Goodbye to the bioassay’ by Jay I. Goodman *et al.*, *Toxicol. Res.*, 2018, **7**, 558–564.



## 

The author would like to add an additional acknowledgement that was omitted from the original article with details of the funding source for this work as follows: “NIH funding: NIH-NIEHS, P42 ES004911, Superfund Program “Environmental, Microbial, and Mammalian Bimolecular Responses to AhR Ligands”, N. E. Kaminski, PI, J. I. Goodman, Core Leader, Training Core; 1 April 2013 through 3 March 2019.”

The Royal Society of Chemistry apologises for these errors and any consequent inconvenience to authors and readers.

